# Adsorption Characteristics and Ecological Risk Control of Multi-Metals in Biogas Slurry Using Blended Cow Dung and Corn Straw Biochar

**DOI:** 10.3390/molecules31162809

**Published:** 2026-08-12

**Authors:** Peng Xiang, Jian Zheng, Zhaokai Yu, Yan Wang

**Affiliations:** 1 Lanzhou University of Technology, Lanzhou 730050, China; 2 Key Laboratory of Multi-Supply System with Solar Energy and Biomass, Gansu Province, Lanzhou 730050, China; 3 Collaborative Innovation Center for Supporting Technology of Northwest Low-Carbon Towns, Gansu Province, Lanzhou 730050, China

**Keywords:** biogas slurry, biochar, multi-metal immobilization, adsorption behavior, potential ecological risk assessment

## Abstract

Biogas slurry can enhance soil fertility, but the heavy metals it contains may pose potential ecological risks to soil-crop systems. To mitigate heavy metal pollution resulting from the application of biogas slurry to soil, this study investigated potential remediation strategies through the use of blended biochar application. In this study, soil incubation experiments were conducted to evaluate the immobilization performance of cow dung biochar (CB), corn straw biochar (SB), and blended biochar (cow dung + corn straw) (C3S7, C5S5, and C7S3) toward Pb, Zn, Ni, Cr, Cu, As, and Cd under different biogas slurry ratios (Z0, Z1:8, and Z1:4). The results concluded that immobilization efficiency consistently followed the order C7S3 ≥ C5S5 > C3S7 > CB ≈ SB, indicating that the blended biochar generally outperformed the two single biochar in the biogas slurry-irrigated soil system. Batch adsorption experiments showed that adsorption of all metals was better described by the pseudo-second-order model (*R*^2^ > 0.94). Isotherm fitting further indicated that Zn, Ni, Cr, Cu, and Cd were better fitted by the Langmuir model, whereas Pb and As were better fitted by the Freundlich model. Physicochemical characterization, scanning electron microscopy–energy-dispersive X-ray spectroscopy (SEM–EDS), and Fourier transform infrared spectroscopy (FTIR) analyses collectively suggested that the superior performance of blended biochar was associated with the integration of mineral-related characteristics from CB and surface chemical properties from SB, which together enhanced the synergistic fixation of coexisting metals. Consistently, biochar application reduced the potential ecological risk index (*RI*) of bioavailable heavy metals in soil, with blended biochar showing lower *RI* values than CB and SB. C7S3 exhibited the best performance in all treatments, highlighting the potential of blended biochar as an effective amendment for mitigating multi-metal pollution risks with biogas slurry utilization.

## 1. Introduction

With the rapid expansion of animal husbandry, the treatment and resource utilization of livestock manure have become central issues in the green transition of agriculture. Anaerobic digestion converts livestock manure into biogas and biogas slurry and therefore contributes to energy recovery, nutrient recycling, and waste reduction [1]. Biogas slurry is rich in plant-available nutrients such as nitrogen (N), phosphorus (P), and potassium (K), and can partially replace chemical fertilizers while improving soil properties [2,3]. However, slurry derived from livestock manure may also contain heavy metals introduced through feed additives and farming practices. These metals can migrate along the “feed–livestock–manure–anaerobic fermentation system–biogas slurry–soil” pathway and accumulate in agricultural soils, thereby posing potential ecological risks [4,5]. Reducing the environmental risk of heavy metals while maintaining the agronomic value of biogas slurry has therefore become an urgent practical issue.

Current technologies for removing heavy metals from wastewater include adsorption, chemical precipitation, ion exchange, membrane separation, electrodialysis, and bioremediation [6,7,8]. Among them, adsorption is widely regarded as an attractive method because of its operational simplicity, high efficiency, and relatively low environmental burden [9,10]. Common adsorbents include zeolite, diatomaceous earth, phosphorus-based passivators, activated carbon, and biochar [11,12,13]. Biochar, a carbon-rich product of biomass pyrolysis, is considered a promising and sustainable amendment because of its porous structure, large specific surface area, and abundant surface functional groups [14]. Biochar can provide different types of adsorption sites and therefore exhibit different affinities for metal ions [15,16]. For example, rice husk biochar shows the highest adsorption capacity for Cu, followed by Cd, Pb, and Zn [17], whereas corn straw biochar has a higher capacity for Pb than for Cd, Cu, and Zn [18]. Cow dung biochar has been reported to adsorb Cu and Pb more strongly than Cd and Ni [19]. Such selectivity implies that single biochar may be less effective in complex multi-metal systems such as actual biogas slurry, where competition among coexisting ions can further reduce adsorption efficiency [20]. In addition, most previous studies have relied on simulated solutions containing one or only a few metals, whereas actual biogas slurry is a chemically complex multi-metal system in which concentration differences and competitive adsorption may weaken the fixation efficiency of biochar and limit its practical application [21].

Cow dung and corn straw are two representative agricultural organic wastes, and their valorization has both environmental and agronomic significance. Corn straw biochar typically shows relatively high carbon content, developed pore structure, large specific surface area, and high cation exchange capacity, which makes it effective for adsorbing metals such as Cr, Cd, and Pb in aqueous systems [22,23,24]. Cow dung biochar, by contrast, is often characterized by porous structure, abundant active groups, and relatively high mineral content, and has been widely used for the adsorption of Cu, Pb, and Cd [25,26,27,28]. These differences suggest that feedstock-dependent heterogeneity in surface chemistry and structure [29]. Accordingly, blending biochar from different feedstocks may improve multi-metal immobilization by combining mineral precipitation, surface complexation, ion exchange, and pore-filling effects within one amendment. However, the ratio-dependent performance of physically blended biochar has been less systematically evaluated in real biogas slurry–irrigated soil systems containing multiple coexisting metals, particularly by integrating adsorption behavior, soil immobilization, and ecological risk assessment.

Against this background, the present study systematically evaluated the effects of blending ratios of cow dung biochar and corn straw biochar on the immobilization of multiple heavy metals in a real biogas slurry-irrigated soil system. CB and SB were separately pyrolyzed and subsequently blended at different mass ratios to obtain C3S7, C5S5, and C7S3. Their performance was first assessed through a soil incubation experiment to determine the immobilization efficiency of Pb, Zn, Ni, Cr, Cu, As, and Cd under different biogas slurry ratios. Batch adsorption experiments, combined with physicochemical characterization, SEM–EDS (scanning electron microscopy–energy-dispersive X-ray spectroscopy), and FTIR (Fourier transform infrared spectroscopy) analyses, were then used to interpret the mechanisms underlying the differences among treatments. Finally, the Hakanson potential ecological risk index was applied to determine whether immobilization of bioavailable heavy metals could be translated into an overall reduction in ecological risk. This study provides experimental evidence for the use of blended biochar to mitigate heavy metal risks during biogas slurry utilization and supports the value-added conversion of cow dung and corn straw.

## 2. Materials and Methods

### 2.1. Preparation of Experimental Materials

The soil used in this experiment was collected from the 0–40 cm tillage layer of farmland in Qilihe District, Lanzhou, Gansu Province, China. The soil was air-dried, visible plant residues were removed, and the material was passed through a 2 mm sieve to obtain a homogeneous sample. Particle size analysis indicated that the soil was a silty loam. The concentrations of heavy metals in the soil are shown in Table 1. CB and SB were produced separately by pyrolysis at 500 °C for 2–3 h under oxygen-limited conditions. The resulting biochar were then physically blended at CB:SB mass ratios of 3:7, 5:5, and 7:3 to obtain C3S7, C5S5, and C7S3, respectively. Heavy metal concentrations in the soil and in each biochar treatment are presented in Table 1.

The biogas slurry was derived from cow dung and obtained from the Gansu Holstein Purebred Dairy Cow Breeding Center, Shuangcheng Town, Wuwei, Gansu Province, China. After anaerobic fermentation, the biogas slurry was settled for two months to stabilize its physicochemical properties. The aged slurry was thoroughly mixed and filtered through four layers of 32-mesh gauze to remove suspended large particles. The biogas slurry was then diluted with deionized water to volume ratios of 1:1, 1:2, 1:4, 1:6, 1:8, 1:10, 1:12, 1:14, and 1:16 (biogas slurry: water). The basic properties and heavy metal concentrations of the stock slurry are presented in Table 2.

### 2.2. Experimental Design and Methods

#### 2.2.1. Soil Incubation Experiment

Various types of biochar (CB, SB, C3S7, C5S5, C7S3) were added at mass ratios of 0%, 2%, and 4%. Three replicates were established for each treatment. The soil and biochar were thoroughly mixed and subjected to three slurry regimes: Z0 (deionized water control), Z1:8 (biogas slurry: water = 1:8, *v*/*v*), and Z1:4 (biogas slurry: water = 1:4, *v*/*v*). The Z1:8 and Z1:4 regimes were selected as representative and agronomically relevant biogas slurry irrigation ratios, as these dilution levels have been commonly used in tomato and biogas slurry irrigation studies and are within the range considered suitable for crop application [30,31]. Moisture was maintained at approximately 80% of field capacity by gravimetric adjustment every two days using deionized water or the corresponding slurry solution to compensate for evaporation. The experiment was conducted at a constant temperature of 25 °C and 60% relative humidity for a duration of 120 days. Samples were collected from each container on days 6, 16, 30, 46, 60, 90, and 120. After sampling, the mixtures were air-dried, ground, and sieved for subsequent testing and analysis.

#### 2.2.2. Adsorption Kinetics Test

A conical flask of 250 mL was mixed with 200 mg of biochar (CB, SB, C3S7, C5S5, and C7S3) and biogas slurry stock solution of 200.0 mL. The pH was maintained at 8.04, corresponding to the measured pH of the original biogas slurry (Table 2), so that pH was not treated as an experimental variable. When necessary, HNO_3_ or NaOH was used to maintain the biogas slurry at pH 8.04. Adsorption kinetics tests were done for the treatments and repeated three times. The conical flask was tightly covered and kept at 25 °C and 180 rpm in a constant temperature shaker for 48 h. Sampling was carried out at 5, 15, 30, 60, 120, 240, 360, 720, 960, 1440, and 2880 min. At every sampling point, a sample was filtered through a 0.22 μm microporous membrane filter and the filtrate was collected to measure heavy metal ion concentrations.

#### 2.2.3. Adsorption Equilibrium Test

Biogas slurry at the different concentrations (stock solution, 1:1, 1:2, 1:4, 1:6, 1:8, 1:10, 1:12, 1:14, and 1:16) and 200 mg of biochar (CB, SB, C3S7, C5S5, and C7S3) were transferred into a 250 mL conical flask correspondingly. The pH of each slurry solution was maintained at 8.04, the original pH of the biogas slurry, to exclude pH variation among dilution treatments. The solution was subsequently shaken for 48 h at the temperature of 25 °C and at a speed of 180 rpm. After oscillation, the suspension was filtered through a 0.22 μm microporous membrane, and the concentrations of heavy metal ions in the filtrate were measured. Each treatment was repeated three times.

### 2.3. Properties Analysis

Using the incineration method, the ash content of the biochar was determined while the pH value of the different biochar treatment materials were measured with a pH meter using a carbon-water mass ratio of 1:20 (FE 20, Mettler-Toledo Instruments, Greifensee, Switzerland). The elemental composition (C, H, O, N and S) of the biochar material characterization was basically studied using dry calcination and element analyzer (Elementar vario EL III, Elementar, Langenselbold, Germany); then the surface morphology and microstructure of the biochar samples were characterized using a scanning electron microscope coupled with energy-dispersive X-ray spectroscopy (SEM–EDS; Hitachi SU8100 cold-field emission scanning electron microscope, Hitachi, Ibaraki, Japan), and EDS elemental mapping was further performed on representative surface regions of CB, SB, C3S7, C5S5, and C7S3 before and after adsorption to visualize the spatial distributions of the seven target metals (Pb, Zn, Ni, Cr, Cu, As, and Cd); cation exchange capacity was studied using hexaamminecobalt trichloride extraction-spectrophotometry; finally the Fourier transform infrared (FTIR) spectra was collected with a Fourier transform infrared spectrometer (Nixolet iS 50, Thermo Fisher Scientific, Waltham, MA, USA) between 4000 and 400 cm^−1^, which was used to characterize functional groups on the different biochar treatment materials before and after the heavy metal ions adsorption from the biogas slurry.

The turbidity, total solids and pH of the biogas slurry were determined using UV-Vis spectrophotometer, drying method and pH meter respectively. The concentrations of Pb, Zn, Ni, Cr, Cu, As, and Cd ions in the biogas slurry and the filtrate during the study were measured through microwave digestion-inductively coupled plasma mass spectrometry (Agilent Technologies 7700 Series ICP-MS, Agilent Technologies, Tokyo, Japan). The concentration of the bioavailable heavy metal ions in the soil was determined using diethylenetriaminepentaacetic acid (DTPA) extraction followed by inductively coupled plasma optical emission spectrometry (PerkinElmer Avio 500 ICP-OES, PerkinElmer, Waltham, MA, USA).

### 2.4. Data Analysis

#### 2.4.1. Passivation Ratio

The passivation ratio of soil heavy metals after application of different amendments was calculated as follows:

(1) $$passivation\;ratio=\left[(C_{1}-C_{2})/C_{1}\right]\times 100\%$$

where *C*_1_ is the concentration of a given bioavailable heavy metal in the control treatment, and *C*_2_ is the concentration of the same metal after application of the biochar amendment.

#### 2.4.2. Adsorption Capacity

The adsorption capacity was calculated using Equation (2):

(2) $$Q_{e}=\frac{\left(C_{0}-C_{e}\right)}{m}\times V$$

where *Q_e_* is the unit adsorption amount at equilibrium (mg g^−1^); *C*_0_ and *C_e_* are the initial and equilibrium concentrations of the heavy metal ions, respectively (mg L^−1^); *V* is the volume of the added biogas slurry solution (mL); and *m* is the mass of the added biochar (mg). Because no biochar free control was included, precipitation/co-precipitation at pH 8.04 was not quantified separately. Therefore, the calculated *Q_e_* values should be regarded as apparent adsorption capacities that may include contributions from both biochar-associated retention and precipitation [32].

#### 2.4.3. Kinetic Adsorption Model

Three commonly used kinetic models were employed to fit the experimental data. The pseudo first order (PFO) model (Equation (3)) assumed that the adsorption rate is proportional to the number of unoccupied sites. The pseudo second order (PSO) model (Equation (4)) assumed that adsorption is dominated by chemisorption and depends on the amount of solute adsorbed at equilibrium and at any given time [33]. The Elovich model (Equation (5)) is commonly used to describe chemisorption on heterogeneous surfaces and assumes that the process is jointly influenced by reaction rate and diffusion effects [34].

(3) $$\ln(Q_{e}-Q_{t})=\ln Q_{e}-k_{1}t$$

(4) $$t/Q_{t}=1/(k_{2}{Q_{t}}^{2})+t/Q_{e}$$

(5) $$Q_{t}=(1/B)\ln(AB)+(1/B)\ln t$$

where *Q_e_* is the unit adsorption amount at equilibrium (mg g^−1^); *Q_t_* is the unit adsorption amount at time t (mg g^−1^); *k*_1_ is the PFO adsorption rate constant (min^−1^); *k*_2_ is the PSO adsorption rate constant (g mg^−1^ min^−1^); *A* is the initial adsorption rate constant (mg g^−1^ min^−1^); *B* is the desorption adsorption rate constant (g mg^−1^); and *t* is the time (min).

#### 2.4.4. Adsorption Isotherm Model

To describe the adsorption behavior of heavy metals in biogas slurry on different biochar, the Langmuir (LIM) and Freundlich (FIM) isotherm models were applied. The Langmuir model (Equation (6)) assumed monolayer adsorption on a homogeneous surface with a finite number of identical active sites and no interaction among adsorbed molecules [35,36,37] (Guo and Wang, 2019; Cai et al., 2021; Wang et al., 2021). It provides Qmax, which is commonly used to evaluate the theoretical adsorption capacity of a material [38]. The Freundlich model (Equation (7)) describes multilayer adsorption on heterogeneous surfaces with different adsorption energies and site types.

(6) $$Q_{e}=\frac{Q_{\max}k_{l}C_{e}}{1+k_{l}C_{e}}$$

(7) $$Q_{e}=k_{f}C_{e}^{\frac{1}{n}}$$

where *Q_e_* is the unit adsorption amount at equilibrium (mg g^−1^); *C_e_* is the equilibrium concentration of the heavy metal ions in the solution (mg L^−1^); *Q_max_* is the theoretical maximum adsorption capacity, (mg g^−1^); *k_l_* is the affinity parameter (L mg^−1^), which is related to the material adsorption strength and the affinity for metal ions; *k_f_* is the Freundlich constant; and n is another constant used to characterize the adsorption strength.

#### 2.4.5. Potential Ecological Risk Evaluation

The potential ecological risk coefficient (*E_i_^r^*) and the overall potential ecological risk index (*RI*) were calculated using the Hakanson method to evaluate the ecological risk associated with heavy metals in the soil system [39,40]. The equations are as follows:

(8) $$C_{f}^{i}=\frac{C_{D}^{i}}{C_{R}^{i}}$$

(9) $$E_{r}^{i}=T_{r}^{i}\times C_{f}^{i}$$

(10) $$RI=\sum_{i=1}^{n}E_{r}^{i}$$

where *i* is the seven bioavailable heavy metals in the soil; *C^i^_f_* is the single heavy metal pollution coefficient; *C^i^_D_* is the average bioavailable heavy metal concentration in the measured soil samples; *C^i^_R_* is the heavy metal background reference value (this study used the bioavailable heavy metals in the Z0B0 treatment soil as the background reference value) [41]; *E_i_^r^* is the potential ecological risk coefficient of heavy metal *i*; *T^i^_r_* is the corresponding toxicity factor for heavy metal *i*; and *RI* is the potential ecological risk index for multiple heavy metals. The biological toxicity coefficients (*T^i^_r_*) for the heavy metals are shown in Table 3.

#### 2.4.6. Random Forest Regression Model Analysis

To assess the relative importance of different factors affecting the passivation rate, a random forest regression (RF) model was constructed. Random forest is an ensemble learning method that integrates multiple decision trees and can effectively capture nonlinear relationships while maintaining good robustness and predictive performance. In this study, the ranger package was used to implement the RF model. The independent variables included biogas slurry ratio, biochar type, biochar blending ratio, and irrigation volume, and the dependent variable was the passivation rate of heavy metals in soil. All input data were derived from laboratory observations across treatments.

### 2.5. Statistical Analysis

Data were analyzed by three-way analysis of variance using SPSS 25.0. Adsorption kinetic and isotherm parameters were fitted in Origin 2018 (Origin Lab, Northampton, MA, USA), and goodness of fit was evaluated using the coefficient of determination (*R*^2^).

## 3. Results

### 3.1. Effect of Different Biochar on the Passivation Rate of Heavy Metal Ions in Biogas Slurry Irrigated Soil

**Figure 1:**
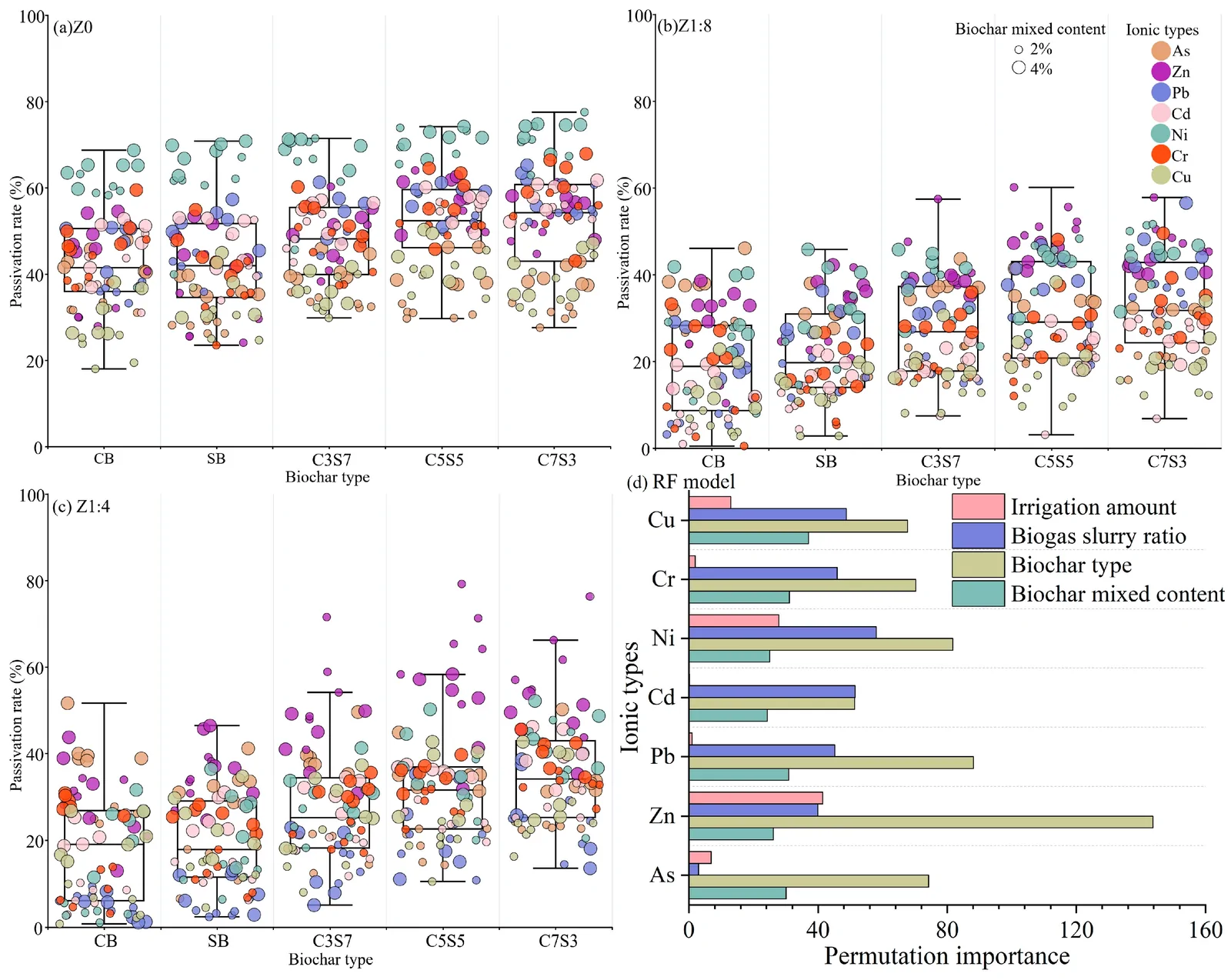
The effect of different biochar treatments on the passivation rate of various heavy metal ions under different biogas slurry ratios and the random forest importance ranking of the main controlling factors.

Under different biogas slurry ratios (Z0, Z1:8, and Z1:4), the passivation rates of heavy metals showed a highly consistent response to biochar type, following the order C7S3 ≥ C5S5 > C3S7 > CB ≈ SB (Figure 1a–c), which indicated that the blended biochar performed better than the two single biochar under all biogas slurry ratio conditions. Their advantage was reflected not only in higher median passivation rates, but also in a broader upper range of values. Under Z0 conditions, the median passivation rates of C7S3, C5S5, and C3S7 were 54.19%, 52.33%, and 48.11%, respectively, representing increases of 16.01–30.68% over CB (41.47%) and 14.77–29.27% over SB (41.92%). Under Z1:8 conditions, the corresponding median values were 31.76%, 29.04%, and 26.84%, which were 42.48–68.59% higher than CB (18.84%) and 36.10–61.07% higher than SB (19.72%). Under Z1:4 conditions, the median passivation rates of C7S3, C5S5, and C3S7 were 34.08%, 31.50%, and 25.17%, respectively, corresponding to increases of 32.41–79.28% over CB (19.01%) and 41.17–91.16% over SB (17.83%). These results indicated that blending biochar improved the overall passivation level in the multi-metal system and also reduced the limitations associated with the selective behavior of single biochar.

Compared with Z0, biogas slurry irrigation generally reduced the passivation rates of the biochar treatments (Figure 1a–c). For example, the median passivation rate of CB decreased from 41.47% under Z0 to 19.01% under Z1:4 and 18.84% under Z1:8, while that of C7S3 decreased from 54.19% to 34.08% and 31.76%, respectively. However, the differences between Z1:4 and Z1:8 were treatment-dependent rather than consistently concentration-dependent. These results suggest that the presence of biogas slurry altered the immobilization performance of the biochar in the multimetal system, whereas biochar type remained the primary factor explaining the variation in passivation performance.

Meanwhile, Zn maintained a relatively high median passivation level under slurry irrigation (44.33% under Z1:4 and 39.91% under Z1:8), whereas metals such as As and Pb generally showed lower values. This indicated that heavy metals responded differently under competitive multi-metal conditions. Permutation importance analysis from the random forest model (Figure 1d) further showed that biochar type was the main factor explaining variation in passivation rate, especially for Zn, followed by biogas slurry ratio and biochar blending ratio, whereas irrigation volume contributed less. Overall, biochar type was the primary determinant of passivation performance, while increasing slurry concentration reduced the overall passivation level and strengthened differences among metals.

### 3.2. Effect of Different Biochar on the Adsorption Kinetics in Biogas Slurry Irrigated Soil

**Figure 2:**
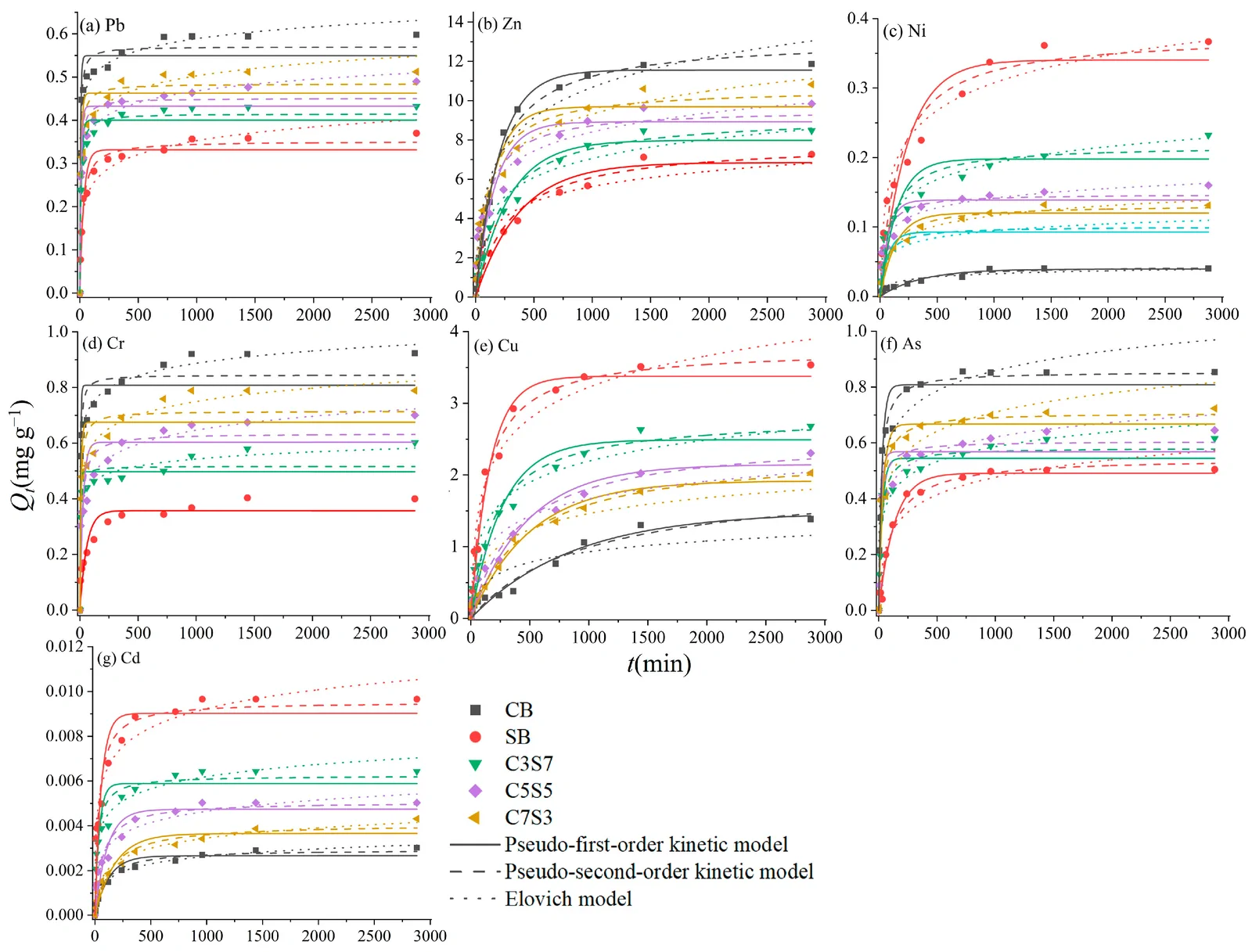
Adsorption kinetics curves.

The adsorption amounts of heavy metal ions in biogas slurry at different contact times are shown in Figure 2. Under all biochar treatments, the adsorption kinetics of all heavy metals followed a similar three-stage pattern, consisting of rapid adsorption, slow adsorption, and eventual equilibrium. To further clarify the adsorption processes of Pb, Zn, Ni, Cr, Cu, As, and Cd on the different biochar, the PFO, PSO, and Elovich models were fitted to the experimental data (Figure 2 and Table 4). The adsorption of all heavy metals was better described by the PSO model, with R^2^ values above 0.94. In addition, the theoretical adsorption capacities estimated by the PSO model agreed well with the measured values, indicating that the PSO model more accurately captured the adsorption behavior of heavy metals in biogas slurry under the different biochar treatments.

The PSO parameters further showed that the *Q_e_* values for Pb, Zn, Cr, and As decreased in the order CB > C7S3 > C5S5 > C3S7 > SB, whereas those for Cu, Cd, and Ni followed the order SB > C3S7 > C5S5 > C7S3 > CB. This indicated that CB had a stronger equilibrium adsorption capacity for Pb, Zn, Cr, and As, while SB showed a stronger equilibrium adsorption capacity for Cu, Cd, and Ni. Under the same biochar treatment, the equilibrium adsorption capacities of heavy metals broadly followed the order Zn > Cu > Cr ~ As > Pb > Ni > Cd, which was generally consistent with their concentration order in biogas slurry (Zn > Cu > As > Cr > Pb > Ni > Cd). This suggested that metals present at higher initial concentrations may have a stronger mass-transfer advantage in the multi-metal system. The *k*_2_ values also showed that the blended biochar generally enhanced the adsorption rates of Zn, Ni, Cr, As, and Cd. In addition, the Elovich model gave relatively high R^2^ values for C3S7, C5S5, and C7S3 (*R*^2^ > 0.92), suggesting that chemisorption on heterogeneous surfaces, including complexation by active functional groups and exchange with adsorbed cations, also contributed to adsorption by the blended biochar.

### 3.3. Effect of Different Biochar on the Adsorption Isotherms in Biogas Slurry Irrigated Soil

**Figure 3:**
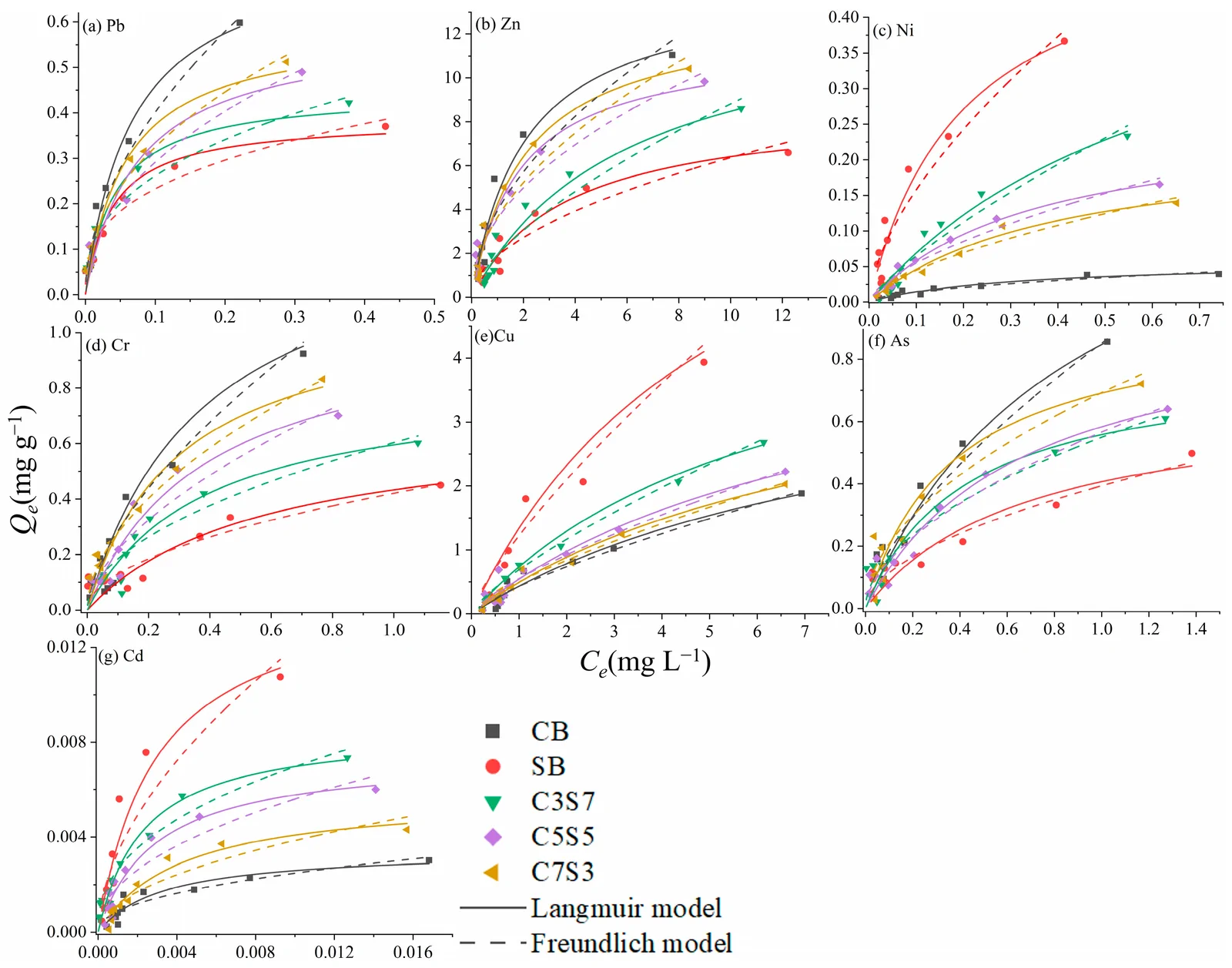
Adsorption isotherms.

Figure 3 shows the adsorption amounts of Pb (0.04–0.80 mg L^−1^), Zn (1.60–18.82 mg L^−1^), Ni (0.04–0.78 mg L^−1^), Cr (0.09–1.60 mg L^−1^), Cu (0.49–8.82 mg L^−1^), As (0.10–1.88 mg L^−1^), and Cd (0.001–0.02 mg L^−1^) by CB, SB, C3S7, C5S5, and C7S3 as a function of equilibrium concentration. As shown, with the increase in the initial concentration of heavy metal ions, the unit adsorption amount of different biochar treatments also increased.

The Langmuir and Freundlich models were used to describe the adsorption isotherms of heavy metals in biogas slurry on the different biochar treatments. The fitted maximum adsorption capacities and model parameters are listed in Table 5. The Langmuir model provided a better fitting performance for Zn, Ni, Cr, Cu, and Cd (*R*^2^ > 0.94), whereas Pb and As were better described by the Freundlich model (*R*^2^ > 0.94). Although the Freundlich model better captured Pb and As adsorption on CB, SB, C3S7, C5S5, and C7S3, the Langmuir model still produced R^2^ values above 0.90, indicating that it also provided a reasonable description of their adsorption behavior.

As shown in Table 5, the theoretical maximum adsorption capacity (*Q_max_*) for Pb, Zn, Cr, and As were higher for CB, C3S7, C5S5, and C7S3 than for SB, whereas the *Q_max_* values for Cu, Cd, and Ni were lower than those of SB. The Langmuir affinity parameter (*k_l_*) was also higher for CB than for the other biochar for Pb, Zn, Cr, and As, while SB showed higher *k_l_* values for Cu, Cd, and Ni. These results further indicated that CB had stronger adsorption potential for Pb, Zn, Cr, and As, whereas SB was more favorable for Cu, Cd, and Ni. The total theoretical maximum adsorption capacities for heavy metals in biogas slurry followed the order C7S3 (23.00 mg g^−1^) > CB (22.12 mg g^−1^) > C5S5 (21.98 mg g^−1^) > C3S7 (20.62 mg g^−1^) > SB (19.22 mg g^−1^). The Freundlich constant (*k_f_*) was higher for SB than for the other biochar for Pb, Zn, Ni, Cu, and Cd, whereas C7S3 showed a higher *k_f_* for Cr and As, suggesting stronger affinity of SB for Pb, Zn, Ni, Cu, and Cd and of C7S3 for Cr and As. In addition, all 1/n values were below 1, indicating favorable adsorption by all biochar treatments.

### 3.4. Effect of Different Biochar on Potential Ecological Risk Assessment in Biogas Slurry Irrigated Soil

**Figure 4:**
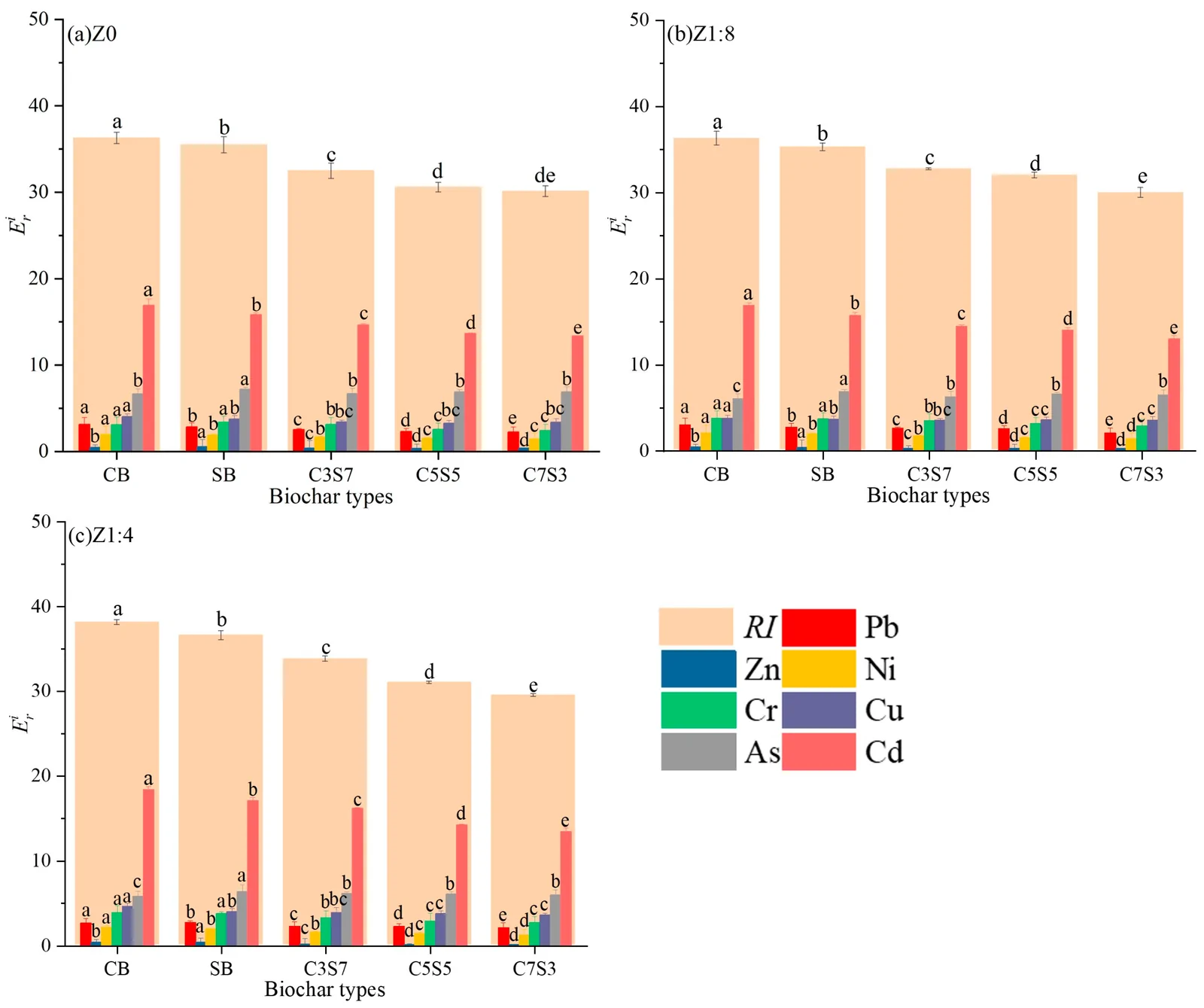
Comparison of the potential ecological risk of soil heavy metals under different biochar treatments. Different lowercase letters (a–e) above bars indicate significant differences among biochar treatments in the potential ecological risk coefficient (*E^i^_r_*) of the same heavy metal or in the overall potential ecological risk index (*RI*) under the same biogas slurry ratio (*p* < 0.05).

Because the concentrations of heavy metals in the bioavailable soil fraction differed substantially in magnitude, simple comparison of total bioavailable metal concentrations would not accurately reflect ecological risk (Li et al.; Xiao et al.). Therefore, Hakanson’s method was used to calculate *E^i^_r_* and *RI* values so that the passivation performance of the different biochar treatments could be evaluated from a risk perspective. In the present study, biochar type significantly (*p* < 0.05) affected the overall potential ecological risk of bioavailable heavy metals in soil. Under Z0 conditions, the *RI* values for CB and SB were 36.29 and 35.50, respectively, whereas the blended biochar reduced *RI* to 32.50 (C3S7), 30.59 (C5S5), and 30.12 (C7S3) (Figure 4). Among these, C7S3 gave the lowest value, reducing *RI* by 16.99% relative to CB. Under Z1:8 conditions, the *RI* values for CB and SB were 36.28 and 35.25, respectively, while the blended biochar reduced *RI* to 32.69 (C3S7), 31.96 (C5S5), and 29.95 (C7S3), with C7S3 reducing *RI* by 17.44% relative to CB. Under Z1:4 conditions, *RI* increased to 38.14 for CB and was 36.59 for SB, whereas the blended biochar still reduced *RI* to 33.80 (C3S7), 31.00 (C5S5), and 29.48 (C7S3). Under this condition, C7S3 lowered *RI* by 22.72% relative to CB. Notably, C7S3 consistently produced the lowest *RI* under all slurry ratios.

The contribution pattern of individual ecological risk coefficients was highly consistent among treatments, following the order Cd > As > (Cu ≈ Cr) > Pb > Ni > Zn (Figure 4). Cd was the dominant contributor to differences in *RI*. Compared with CB, C7S3 reduced the *E^i^_r_* value of Cd from 16.90, 16.91, and 18.41 under Z0, Z1:8, and Z1:4 to 13.36, 13.02, and 13.45, respectively, corresponding to reductions of 20.96–26.92%. At the same time, C7S3 also reduced the risk contribution of secondary metals such as Pb, Ni, and Cr. For example, under Z1:4, the *E^i^_r_* value of Ni decreased from 2.18 to 1.29 and that of Cr from 3.91 to 2.75, leading to the largest decrease in *RI*. By contrast, the single biochar treatments showed more selective effects on individual metals. Relative to SB, CB consistently reduced *E^As^_r_* by 7.56–12.20% across biogas slurry ratios, but increased *E^Cu^_r_* by 4.49–15.11%. SB showed the opposite tendency, with lower *E^Cu^_r_* but higher *E^As^_r_*. The blended biochar, however, reduced both *E^As^_r_* and *E^Cu^_r_* at the same time. Relative to SB, *E^As^_r_* decreased by 3.73–8.55% and *E^Cu^_r_* decreased by 0.97–12.77% under Z0, Z1:8, and Z1:4. Relative to CB, the blended biochar also reduced *E^Cu^_r_* by 5.23–21.81% under all slurry ratios. These results indicated that the blended biochar lowered *RI* not by selectively suppressing one metal, but by jointly reducing several key risk-dominant metals.

### 3.5. Physical and Chemical Properties of Biochar

As shown in Table 6, the physicochemical properties differed markedly among the five biochar treatments. CB had the highest specific surface area, pore volume, ash content, and pH, whereas its average pore size was the smallest. In contrast, SB had the lowest specific surface area, pore volume, ash content, and pH, but the largest average pore size. Compared with CB, SB also showed higher C, H, and O contents, as well as higher H/C, O/C, and CEC values. The three blended biochar (C3S7, C5S5, and C7S3) generally showed intermediate values between those of CB and SB. As the proportion of CB increased, specific surface area, pore volume, ash content, and pH increased, whereas average pore size decreased. In addition, C content increased from C3S7 to C7S3, while N content decreased slightly. Overall, the physicochemical properties of the blended biochar varied systematically with blending ratio.

### 3.6. Surface Morphology of Biochar Before and After Heavy Metal Adsorption

**Figure 5:**
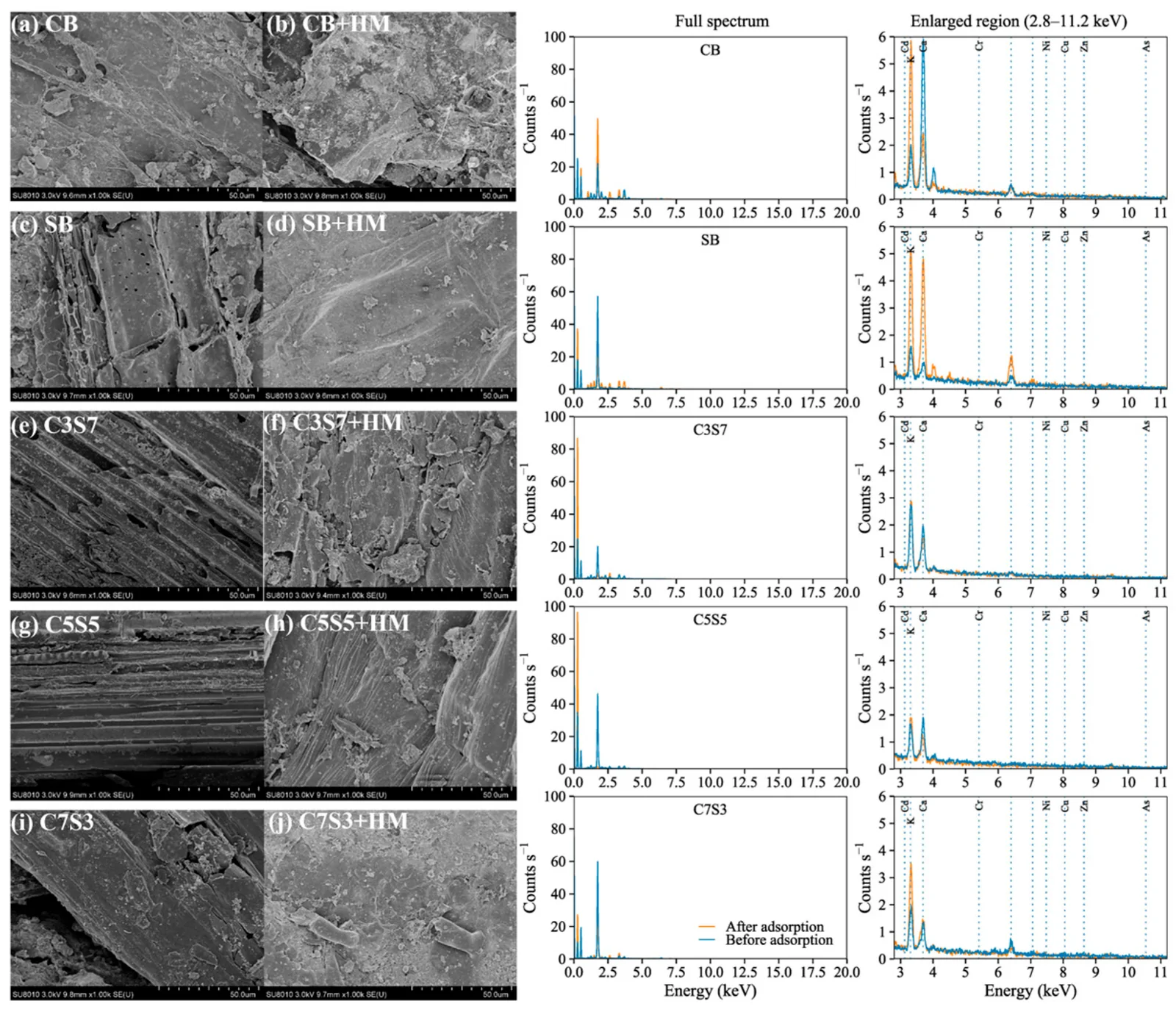
SEM images and EDS spectra of different biochar before and after adsorption of heavy metals from biogas slurry.

As shown in Figure 5, the different biochar exhibited distinct surface morphological and elemental characteristics before and after adsorption of heavy metals from biogas slurry. Before adsorption, CB showed an irregular clumped structure with a relatively rough surface, whereas SB displayed a more intact fibrous carbon skeleton with clearer pore channels. C3S7, C5S5, and C7S3 generally showed intermediate morphological features. After adsorption, particle deposition appeared on the surfaces of all biochar to varying extents, although the patterns differed among treatments. CB showed more pronounced surface coverage and partial pore blockage. In contrast, SB retained a relatively clear carbon framework and pore structure despite increased surface attachment. The blended biochar also exhibited obvious surface depositions after adsorption, but their pore structures remained comparatively distinguishable, especially in C5S5 and C7S3.

Consistent with the SEM observations, the corresponding EDS spectra showed clear changes in the characteristic energy regions associated with K, Ca, Cd, Cr, Ni, Cu, Zn, As, and Pb after adsorption, providing evidence for the retention of biogas slurry-derived inorganic components and heavy metals on the biochar surfaces. To further visualize the spatial distribution of the target metals, EDS elemental mapping was performed for CB, SB, C3S7, C5S5, and C7S3 before and after adsorption. The elemental maps of Pb, Zn, Ni, Cr, Cu, As, and Cd showed heterogeneous spatial distributions of metal-associated signals across the analyzed biochar surface regions (Figures S1–S5), providing complementary visual evidence for the surface retention of multiple metals. Among the different treatments, CB exhibited more pronounced changes in inorganic-element-related signals after adsorption, whereas SB showed enhanced elemental signals while retaining a relatively clear surface structure. C7S3 combined evident surface deposition with relatively good structural preservation, suggesting a more favorable balance between the retention of biogas slurry-derived components and preservation of the biochar structure.

### 3.7. FTIR Analysis of Biochar Before and After Heavy Metal Adsorption

**Figure 6:**
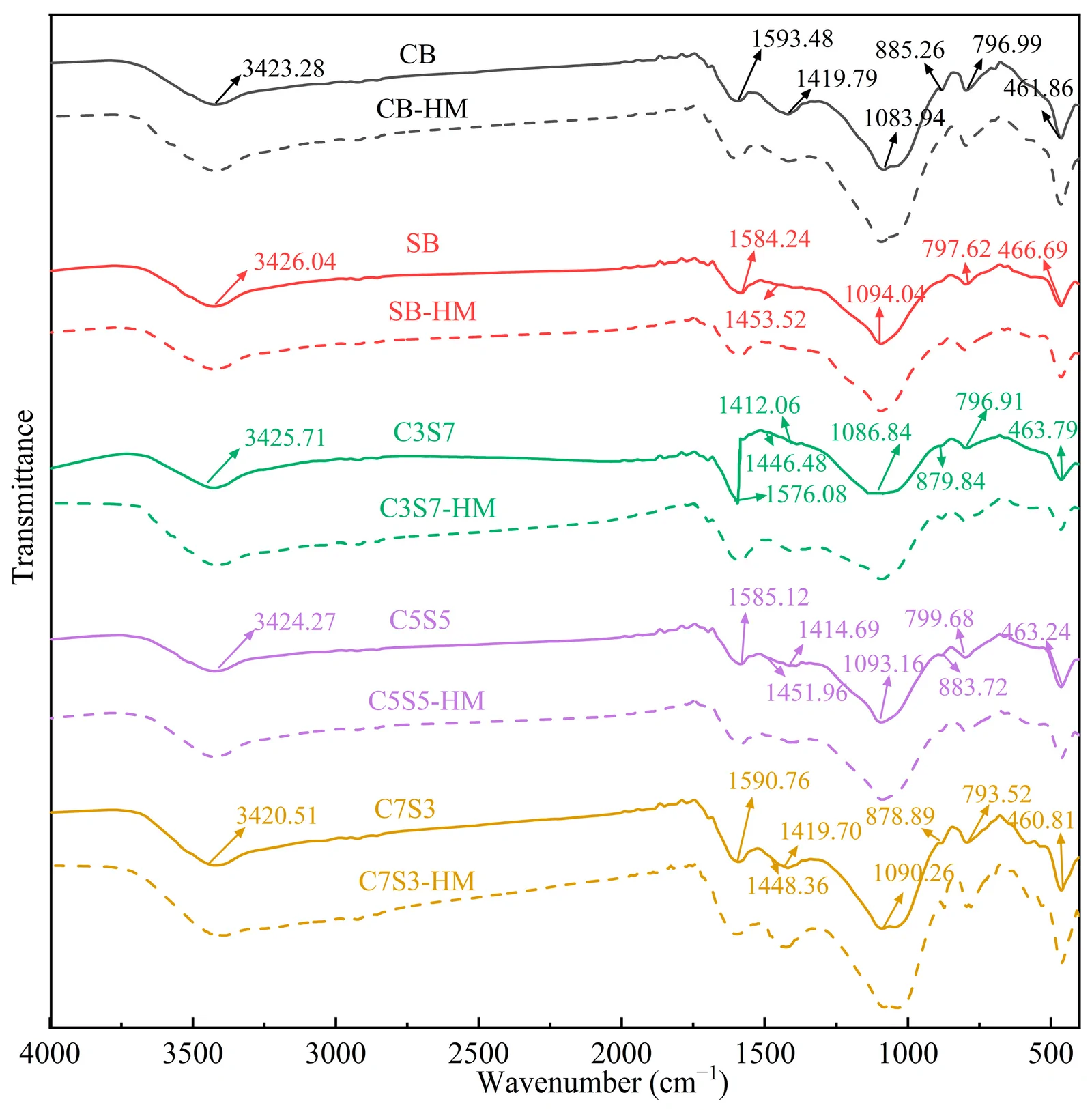
Fourier transform infrared spectra for the biochar treatments before and after heavy metal adsorption.

Figure 6 compared the FTIR spectra of CB, SB, C3S7, C5S5, and C7S3 before and after adsorption of heavy metals from biogas slurry. After adsorption, the –OH stretching vibration band at 3420.51–3426.04 cm^−1^ shifted for all treatments. The characteristic peaks at 1576.08–1593.48 cm^−1^, including COO^−^ antisymmetric stretching, aromatic C=C/C=O stretching, and –NO_2_ asymmetric stretching, increased in intensity after adsorption. A distinct CO_3_^2−^ absorption band appeared in the 1410–1450 cm^−1^ region for CB and the CB-containing biochar (C3S7, C5S5, and C7S3), and the CB peak shifted to 1419.79 cm^−1^ after adsorption. In addition, carbonate-related peaks were observed at 878.89–885.26 cm^−1^. For SB and the SB-containing treatments (SB, C3S7, C5S5, and C7S3), a –CH_2_– stretching vibration peak appeared in the 1446.48–1453.52 cm^−1^ region. After adsorption, the peaks at 1083.94–1094.04 cm^−1^, assigned to C–O–C and Si–O stretching, shifted, and the intensity of the aromatic-related peaks near 793.52–799.68 cm^−1^ weakened or disappeared. An Si–O–Si absorption peak also appeared at 460.81–466.69 cm^−1^.

## 4. Discussion

Under different biogas slurry ratios, the biochar treatments differed markedly in their ability to immobilize heavy metals in the soil incubation system. Overall, the blended biochar outperformed the two single biochar, with C7S3 showing the best overall passivation performance. Passivation generally declined with increasing biogas slurry concentration, likely because stronger competition among coexisting ions reduced the availability of effective sorption and fixation sites in the multi-metal system. Nevertheless, the blended biochar maintained a clear advantage over CB and SB, indicating greater immobilization capacity under the chemically complex conditions of actual biogas slurry. The relatively high passivation of Zn across slurry ratios may be attributable to its higher initial concentration and the resulting mass-transfer advantage under competitive conditions. Combined adsorption and characterization results further indicate that this superior performance did not arise from a single dominant property, but from the complementary contributions of two separately prepared biochar. CB mainly provided mineral-associated and alkaline components that favored pH increase, ion exchange, and mineral precipitation, whereas SB contributed more oxygen-containing surface binding sites. Their post-pyrolysis blending therefore integrated these complementary properties within one amendment and enhanced the overall immobilization of coexisting metals in the biogas slurry–soil system [42,43,44]. It should be noted that co-pyrolysis can induce interactions between different feedstocks during thermal conversion and consequently alter the pore structure, surface functional groups, and heavy-metal stabilization behavior of the resulting biochar [45,46]. Therefore, direct comparison between co-pyrolysis and post-pyrolysis physical blending warrants further investigation.

These treatment differences were closely associated with biochar properties. CB had the highest ash content, pH, specific surface area, and pore volume, indicating a stronger contribution from mineral phases and alkaline constituents. Such properties are commonly linked to metal retention through precipitation or co-precipitation with carbonate- and phosphate-containing phases, particularly for Pb and Zn [47,48]). In contrast, SB showed higher O/C, H/C, and CEC values, suggesting a greater contribution from oxygen-containing functional groups and ion-exchange capacity. These features are often associated with the immobilization of Cu, Cd, and Ni through surface complexation and ion exchange [43,47]. The blended biochar showed intermediate values for most properties, and increasing the proportion of CB increased ash content, pH, specific surface area, and pore volume while decreasing average pore size. The systematic changes in physicochemical properties with blending ratio indicate that the relative contributions of CB-derived mineral and alkaline components and SB derived oxygen containing functional groups and ion exchange sites were correspondingly adjusted. This complementary combination likely altered the availability and nature of binding domains for different metals, thereby regulating the metal-specific adsorption behavior of the blended biochar and contributing to the better overall performance of C7S3 [49,50].

The characterization results supported this interpretation. After adsorption, CB showed pronounced particle deposition and partial pore blockage, whereas SB retained a relatively clear carbon framework and pore structure despite increased surface attachment. The blended biochar also showed obvious surface deposition, but their pore structures remained comparatively distinguishable, especially in C5S5 and C7S3. Corresponding EDS spectra showed changes in the characteristic energy regions of K, Ca, Cd, Cr, Ni, Cu, Zn, As, and Pb, indicating that slurry-derived inorganic components and heavy metal species were retained on the biochar surfaces to different extents. EDS elemental maps further showed heterogeneous spatial distributions of signals assigned to Pb, Zn, Ni, Cr, Cu, As, and Cd across the analyzed biochar surface regions, indicating that multiple coexisting metals were retained at different surface domains of the biochar. The FTIR results further supported the involvement of surface functional groups and mineral-related structures in metal retention. Shifts in the –OH band suggested the involvement of hydroxyl-containing groups in metal binding [51], whereas shifts in the C–O–C/Si–O region indicated that oxygen-containing functional groups and mineral-related structures also participated in adsorption [52]. In CB and the CB-containing blended biochar, changes in carbonate-related peaks after adsorption are likewise consistent with possible carbonate-associated fixation. It should also be recognized that the experiments were conducted at the native pH of the biogas slurry (pH 8.04), under which abiotic precipitation or co-precipitation may contribute to the removal of some dissolved metals [32]. The increased intensity near 1576.08–1593.48 cm^−1^ may reflect changes in aromatic or oxygen-containing structures during adsorption [53]. Taken together, the physicochemical, SEM–EDS, and FTIR results support a multi-pathway retention process involving interactions with oxygen containing functional groups, mineral associated retention, ion exchange, surface accumulation, and possible carbonate associated fixation, with their relative contributions varying among the different biochar treatments [54].

The adsorption kinetics and isotherm results further supported this site-complementary mechanism. The adsorption kinetics of all metals under the different biochar treatments followed a similar three-stage pattern: rapid adsorption, slow adsorption, and equilibrium, which is consistent with the findings of Yan et al. [23]. This pattern is mainly attributable to the large number of available active sites on the biochar surface at the initial stage, which resulted in a rapid increase in adsorption capacity. As these sites gradually became occupied, the system entered a slower adsorption phase, during which the adsorption rate decreased markedly before gradually approaching equilibrium [24]). Adsorption of heavy metal ions from biogas slurry by all biochar treatments was better described by the PSO kinetic model (R^2^ > 0.94), which was consistent with previous studies [55,56]. Because the PSO model is commonly associated with chemisorption-dominated processes, the results suggested that surface reactions played an important role in metal uptake [57,58]. Adsorption capacity also varied among biochar and metals. CB showed higher equilibrium capacities for Pb, Zn, Cr, and As, whereas SB showed higher capacities for Cu, Cd, and Ni, indicating that differences in biochar properties led to different site types and thus selective binding behavior in the multi-metal system, consistent with previous reports [15,16]. Under the same biochar treatment, the adsorption order broadly followed the initial concentration order of the metals in biogas slurry, namely Zn > Cu > Cr ~ As > Pb > Ni > Cd, suggesting that concentration-dependent mass transfer also influenced adsorption in the competitive system [59]. This trend also helped explain the incubation results, in which Zn maintained relatively high passivation across slurry ratios. In addition, the isotherm results showed that Zn, Ni, Cr, Cu, and Cd were better fitted by the Langmuir model, whereas Pb and As were better fitted by the Freundlich model, indicating clear differences in adsorption behavior among metals [60]. These contrasting fitting patterns suggest that the contribution of finite-site adsorption and heterogeneous surface interactions varied among metals. Accordingly, the overall performance of C7S3 likely resulted from a favorable balance among multiple complementary retention pathways rather than from a single dominant adsorption mechanism.

The Hakanson ecological risk assessment further showed that biochar application reduced the overall risk associated with bioavailable heavy metals in soil, with the blended biochar generally producing lower *RI* values than the single biochar. Among them, C7S3 consistently showed the lowest *RI* under all slurry ratios, indicating a more stable overall risk-reduction effect. The contribution pattern of individual metals was similar among treatments, with Cd making the largest contribution to *RI*, followed by As, and then Cu and Cr. This agreed with previous reports that the ecological risk associated with slurry- or digestate-derived heavy metals is often dominated by a small number of high-weight metals [61]. Compared with the single biochar, the blended biochar did not simply reduce the contribution of one metal; rather, they lowered the risk contributions of several key metals simultaneously. This coordinated reduction in multiple risk-dominant metals likely explains why the blended biochar, particularly C7S3, performed better overall in the soil system.

## 5. Conclusions

(1) Under different biogas slurry ratios, heavy metal passivation followed the trend C7S3 ≥ C5S5 > C3S7 > CB ≈ SB. Overall, the blended biochar performed better than CB and SB, with C7S3 showing the best passivation effect. As biogas slurry concentration increased, the overall passivation rate declined.
(2) In the kinetic adsorption analysis, the PSO model provided a better fit than the PFO model for the adsorption of heavy metal ions from biogas slurry by different biochar (*R*^2^ > 0.94). Isotherm fitting further showed that Zn, Ni, Cr, Cu, and Cd were better fitted by the Langmuir model, whereas Pb and As were better fitted by the Freundlich model.
(3) CB exhibited a higher specific surface area and pore volume, while SB demonstrated a greater abundance of oxygen-containing functional groups and a higher cation exchange capacity. The blended biochar integrated the complementary physicochemical characteristics of CB and SB, and variation in the blending ratio systematically altered these properties, thereby influencing the adsorption behavior of multiple heavy metals in biogas slurry.
(4) Biochar application reduced the *RI* of bioavailable heavy metals in soil. The blended biochar produced lower ecological risk than CB and SB, and C7S3 showed the best performance in all treatments.

## Figures and Tables

**Table 1:** Heavy metal ion contents in soil and biochar (mg kg^−1^).

	Cr	Ni	Cu	Zn	As	Cd	Pb
Soil	34.56	33.17	64.65	204.18	11.27	0.32	16.57
CB	57.15	25.26	97.53	353.08	3.64	0.19	9.69
SB	6.90	5.89	12.70	67.82	6.78	0.03	3.04
C3S7	28.69	16.58	42.36	146.31	8.88	0.06	4.49
C5S5	36.94	19.29	78.77	281.82	9.33	0.17	7.59
C7S3	39.98	19.60	69.81	257.91	7.21	0.12	8.08

**Table 2:** Basic properties of the biogas slurry stock solution.

Turbidity	Total Solids (g L^−1)^	pH	Pb (mg L^−1^)	Zn (mg L^−1^)	Ni (mg L^−1^)	Cr (mg L^−1^)	Cu (mg L^−1^)	As (mg L^−1^)	Cd (mg L^−1^)
30,154	49.53	8.04	0.8	18.82	0.78	1.6	8.816	1.88	0.02

**Table 3:** Biological toxicity coefficients (*T^i^_r_*).

	Pb	Zn	Ni	Cr	Cu	As	Cd
*T^i^_r_*	5	1	5	5	5	10	30

**Table 4:** Kinetic model fitting parameters.

Heavy Metal Ions	Biochar Types	Pseudo-First-Order Kinetic Model			Pseudo-Second-Order Kinetic Model			Elovich Model		
		*Q_e_* (mg g^−1^)	*k*_1_ (min^−1^)	*R* ^2^	*Q_e_* (mg g^−1^)	*k*_2_ (g mg^−1^ min^−1^)	*R* ^2^	*B* (g mg^−1^)	*A* (mg g^−1^ min^−1^)	*R* ^2^
Pb	CB	0.4393	0.1458	0.9354	0.5403	0.3975	0.9775	6.6206	25.3039	0.9429
	SB	0.2216	0.0303	0.9424	0.3019	0.1235	0.9842	0.1009	21.8144	0.9105
	C3S7	0.4004	0.0781	0.8693	0.4453	0.3306	0.9472	6.2659	28.2295	0.9555
	C5S5	0.4527	0.0888	0.8350	0.4810	0.3213	0.9884	6.0984	25.5478	0.9379
	C7S3	0.4831	0.0903	0.8353	0.5246	0.2861	0.9684	5.2603	23.3073	0.9452
Zn	CB	10.5572	0.0049	0.9862	11.046	0.0004	0.9921	0.2691	0.4489	0.9536
	SB	6.8473	0.0027	0.9381	8.2314	0.0004	0.9607	0.1667	0.8883	0.9247
	C3S7	7.9810	0.0035	0.9527	9.2563	0.0005	0.9762	0.2135	0.7117	0.9474
	C5S5	8.9199	0.0060	0.8513	9.9732	0.0011	0.9425	0.5690	0.7146	0.9247
	C7S3	9.6913	0.0065	0.9181	10.8231	0.0009	0.9651	0.4162	0.5906	0.9467
Ni	CB	0.0204	0.0028	0.9024	0.0246	0.0855	0.9424	0.0012	60.7723	0.9037
	SB	0.2404	0.0043	0.9187	0.3964	0.0168	0.9570	0.0122	17.4164	0.9537
	C3S7	0.2079	0.0068	0.9118	0.2775	0.0453	0.9577	0.0096	29.3663	0.9352
	C5S5	0.1287	0.0142	0.8196	0.1874	0.1586	0.9433	0.0231	49.8317	0.9288
	C7S3	0.1101	0.0072	0.9361	0.1418	0.0779	0.9758	0.0058	48.4164	0.9606
Cr	CB	0.8084	0.1818	0.8523	0.8450	0.2851	0.9830	6.7227	15.6789	0.9250
	SB	0.3874	0.0174	0.8872	0.4208	0.0701	0.9478	0.0645	19.3075	0.9148
	C3S7	0.4476	0.1951	0.8726	0.5867	0.5576	0.9568	8.6331	27.2648	0.9307
	C5S5	0.5235	0.0379	0.7667	0.6948	0.0965	0.9658	0.5635	13.8652	0.9428
	C7S3	0.6148	0.1034	0.7790	0.7648	0.1830	0.9577	3.3986	14.4050	0.9348
Cu	CB	1.1838	0.0012	0.9643	1.3525	0.0005	0.9591	0.0165	4.6738	0.8820
	SB	3.7771	0.0062	0.9802	4.1604	0.0021	0.9896	0.1041	1.5828	0.9679
	C3S7	2.4899	0.0037	0.9412	3.6138	0.0018	0.9689	0.0734	2.3483	0.9582
	C5S5	2.1474	0.0021	0.9551	3.2330	0.0010	0.9741	0.0402	2.8406	0.9299
	C7S3	1.6194	0.0020	0.9766	2.9202	0.0009	0.9865	0.0309	3.1302	0.9323
As	CB	0.7880	0.0358	0.9532	0.8546	0.0602	0.9846	0.3895	9.5759	0.9397
	SB	0.2317	0.0075	0.9824	0.3462	0.0173	0.9777	0.0176	11.0436	0.9281
	C3S7	0.4450	0.0336	0.9182	0.5824	0.0760	0.9594	0.1572	12.9999	0.9526
	C5S5	0.5680	0.0491	0.8880	0.6955	0.1041	0.9398	0.2941	13.3253	0.9297
	C7S3	0.6273	0.0271	0.9145	0.7679	0.0560	0.9483	0.1254	10.0463	0.9101
Cd	CB	0.0012	0.0080	0.9426	0.0020	3.6467	0.9801	0.0001	2073.7197	0.9375
	SB	0.0090	0.0176	0.8652	0.0118	3.0252	0.9655	0.0023	818.4560	0.9047
	C3S7	0.0059	0.0259	0.8757	0.0092	6.3333	0.9878	0.0021	1272.4062	0.9249
	C5S5	0.0047	0.0092	0.8892	0.0087	2.9323	0.9807	0.0004	1373.6852	0.9458
	C7S3	0.0037	0.0056	0.9286	0.0063	1.8032	0.9810	0.0001	1527.8531	0.9771

**Table 5:** Isotherm model fitting parameters.

Heavy Metal Ions	Biochar Types	Langmuir Model			Freundlich Model		
		*Q_max_* (mg g^−1^)	*k_l_* (L mg^−1^)	*R* ^2^	*k_f_* (mg g^−1^)	1/*n*	*R* ^2^
Pb	CB	0.7093	14.7572	0.9528	1.3541	0.5281	0.9666
	SB	0.3166	25.4473	0.9207	0.5162	0.3454	0.9508
	C3S7	0.4469	23.3941	0.9390	0.6326	0.3834	0.9660
	C5S5	0.5843	13.4166	0.9403	0.8585	0.4691	0.9798
	C7S3	0.6215	17.1346	0.9707	0.9308	0.4570	0.9831
Zn	CB	14.1039	0.4860	0.9597	3.9625	0.5291	0.8974
	SB	8.2634	0.2723	0.9530	1.8918	0.5271	0.9039
	C3S7	11.9566	0.1668	0.9111	1.9377	0.6580	0.8850
	C5S5	12.6457	0.5351	0.9546	3.5821	0.4792	0.9420
	C7S3	13.9186	0.4849	0.9804	3.6524	0.5187	0.9327
Ni	CB	0.0576	3.3381	0.9415	0.0507	0.5524	0.9283
	SB	0.5403	5.0362	0.9474	0.6593	0.6206	0.9238
	C3S7	0.4381	1.4712	0.9617	0.3984	0.7876	0.9378
	C5S5	0.3629	2.8396	0.9859	0.2375	0.6359	0.9597
	C7S3	0.3271	2.5454	0.9765	0.1927	0.6328	0.9589
Cr	CB	1.401	2.6401	0.9530	1.2007	0.6254	0.9216
	SB	0.6806	1.7484	0.9466	0.4208	0.5120	0.9256
	C3S7	0.7187	2.6136	0.9070	0.6032	0.5146	0.8922
	C5S5	1.2578	2.5909	0.9158	0.8280	0.5820	0.8931
	C7S3	1.3165	3.3423	0.9227	0.9562	0.5375	0.9025
Cu	CB	4.2641	0.1131	0.9588	0.4319	0.7697	0.9517
	SB	8.7185	0.1818	0.9113	1.2237	0.7798	0.8796
	C3S7	6.3421	0.1598	0.9512	0.6900	0.7593	0.9384
	C5S5	5.8627	0.1259	0.9464	0.5511	0.7456	0.9465
	C7S3	5.4673	0.1240	0.9798	0.4992	0.7496	0.9795
As	CB	1.5854	1.1462	0.9037	0.8511	0.6655	0.9141
	SB	0.6828	1.4729	0.9115	0.3937	0.5375	0.9417
	C3S7	0.7126	2.3745	0.9137	0.5493	0.5252	0.9219
	C5S5	1.2674	1.5191	0.9208	0.5661	0.5662	0.9397
	C7S3	1.3469	2.7026	0.9066	0.6927	0.5239	0.9214
Cd	CB	0.0034	2.2863	0.9169	0.0202	0.4554	0.8584
	SB	0.0147	8.7678	0.9202	0.1536	0.5540	0.8553
	C3S7	0.0086	6.1684	0.9455	0.0484	0.4211	0.9543
	C5S5	0.0074	4.7275	0.9439	0.0493	0.4724	0.8675
	C7S3	0.0058	3.7768	0.9543	0.0661	0.3273	0.8743

**Table 6:** Physical and chemical properties of the biochar.

Biochar Type	Specific Surface Area (m ^2^ g^−1^)	Pore Volume (cm^−3^ g^−1^)	Average Adsorption Pore Size (nm)	Ash (%)	N (%)	C (%)	H (%)	O (%)	O/C	H/C	CEC (cmol kg^−1^)	pH
CB	155.19	0.08	6.61	74.58	0.70	37.38	0.95	8.22	0.16	0.31	3.20	9.90
SB	66.87	0.05	8.39	7.23	0.67	50.33	2.13	11.80	0.18	0.51	14.54	9.54
C3S7	90.28	0.06	7.58	27.44	0.65	39.26	1.12	9.23	0.18	0.34	11.73	9.75
C5S5	113.56	0.07	7.23	29.56	0.62	42.56	1.88	9.76	0.17	0.53	10.00	9.77
C7S3	134.59	0.07	7.12	32.56	0.55	44.11	1.76	12.30	0.21	0.48	7.21	9.79

## Data Availability

Data will be made available on request.

## Supplementary Materials

The following supporting information can be downloaded at: https://www.mdpi.com/article/10.3390/molecules31162809/s1, Figure S1: EDS elemental maps of Pb, Zn, Ni, Cr, Cu, As, and Cd on CB before and after adsorption of heavy metals from biogas slurry; Figure S2: EDS elemental maps of Pb, Zn, Ni, Cr, Cu, As, and Cd on SB before and after adsorption of heavy metals from biogas slurry; Figure S3: EDS elemental maps of Pb, Zn, Ni, Cr, Cu, As, and Cd on C3S7 before and after adsorption of heavy metals from biogas slurry; Figure S4: EDS elemental maps of Pb, Zn, Ni, Cr, Cu, As, and Cd on C5S5 before and after adsorption of heavy metals from biogas slurry; Figure S5: EDS elemental maps of Pb, Zn, Ni, Cr, Cu, As, and Cd on C7S3 before and after adsorption of heavy metals from biogas slurry.
