# Supplementary material for: Adsorption Characteristics and Ecological Risk Control of Multi-Metals in Biogas Slurry Using Blended Cow Dung and Corn Straw Biochar
Source: Molecules. 2026 Aug 12;31(16):2809. doi: 10.3390/molecules31162809 (PMC13515637; doi:10.3390/molecules31162809)
Supplement: Supplementary file 1 [file molecules-31-02809-s001.zip › molecules-4482415-supplementary.pdf]

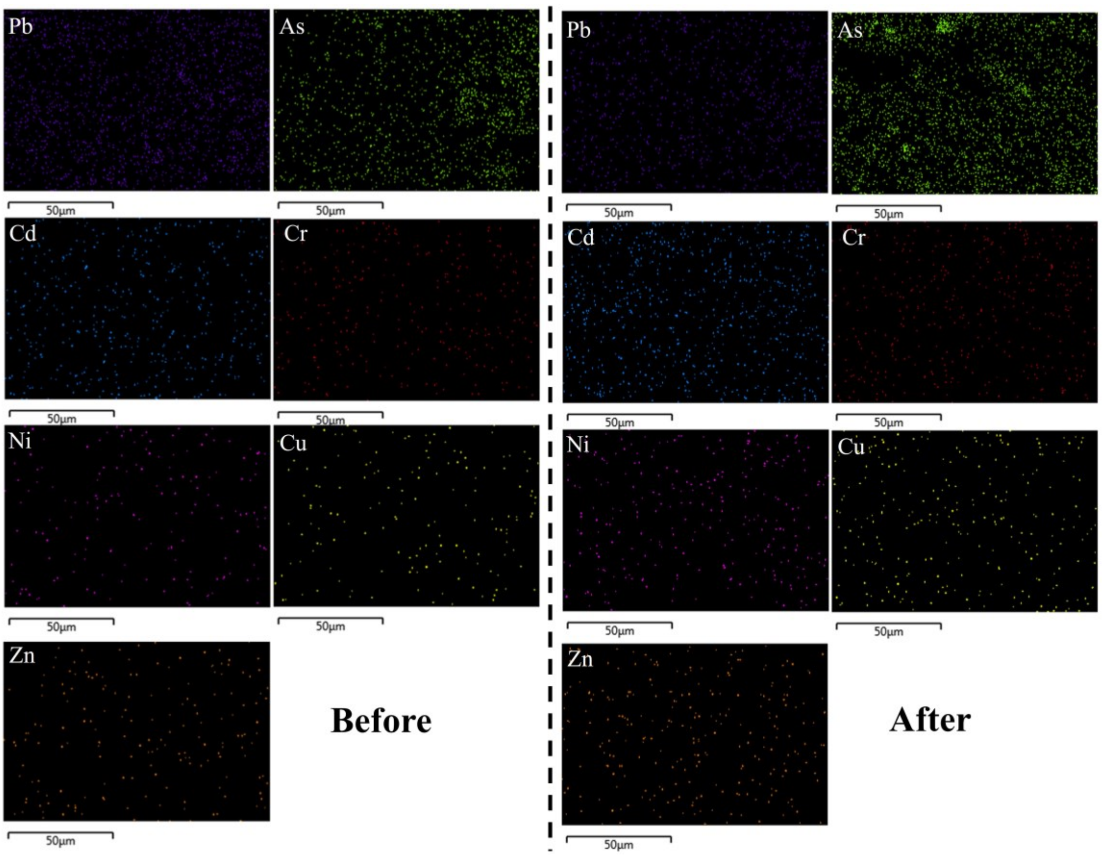

Figure S1. EDS elemental maps of Pb, Zn, Ni, Cr, Cu, As, and Cd on CB before and after adsorption of heavy metals from biogas slurry

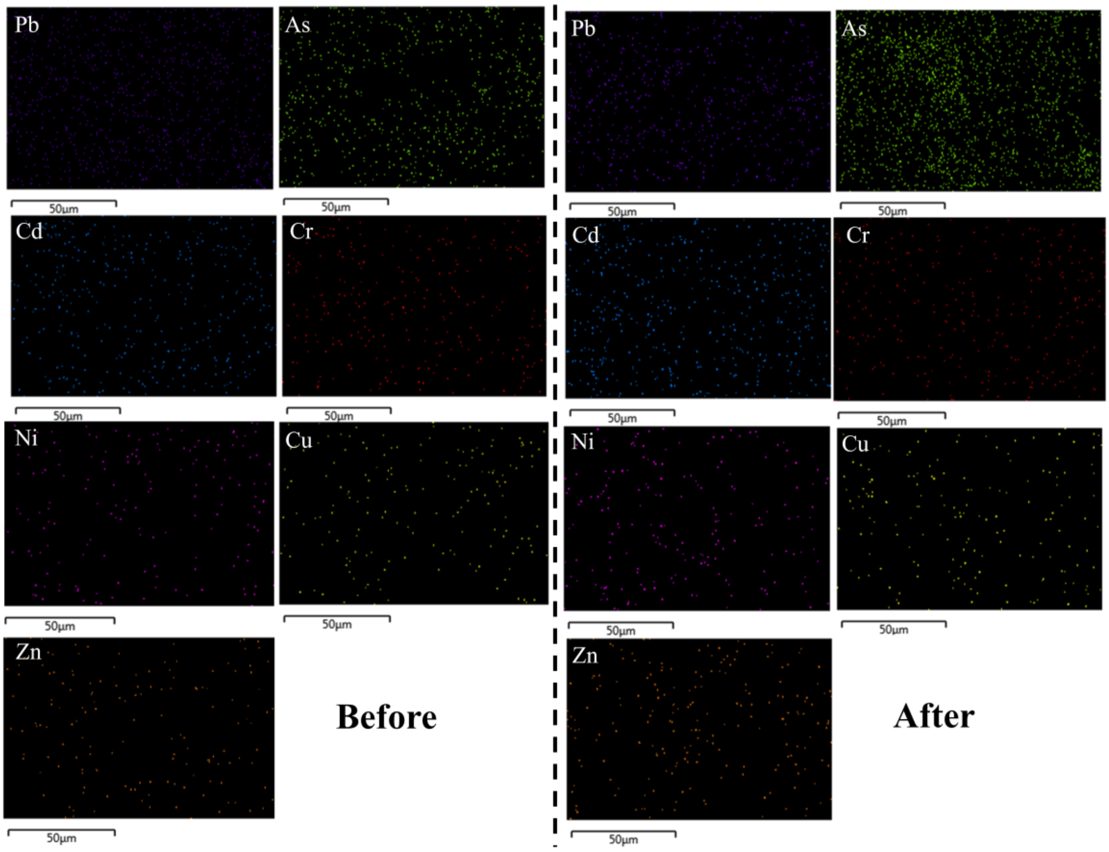

Figure S2. EDS elemental maps of Pb, Zn, Ni, Cr, Cu, As, and Cd on SB before and after adsorption of heavy metals from biogas slurry

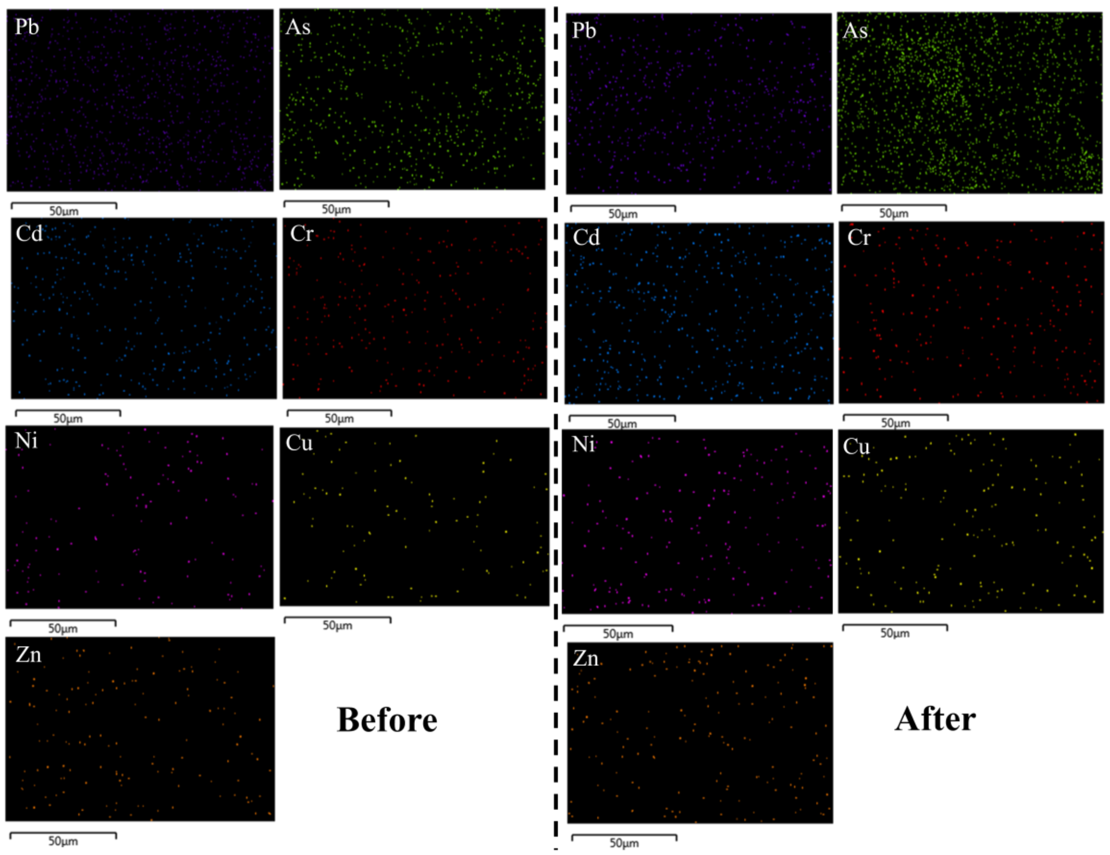

Figure S3. EDS elemental maps of Pb, Zn, Ni, Cr, Cu, As, and Cd on C3S7 before and after adsorption of heavy metals from biogas slurry

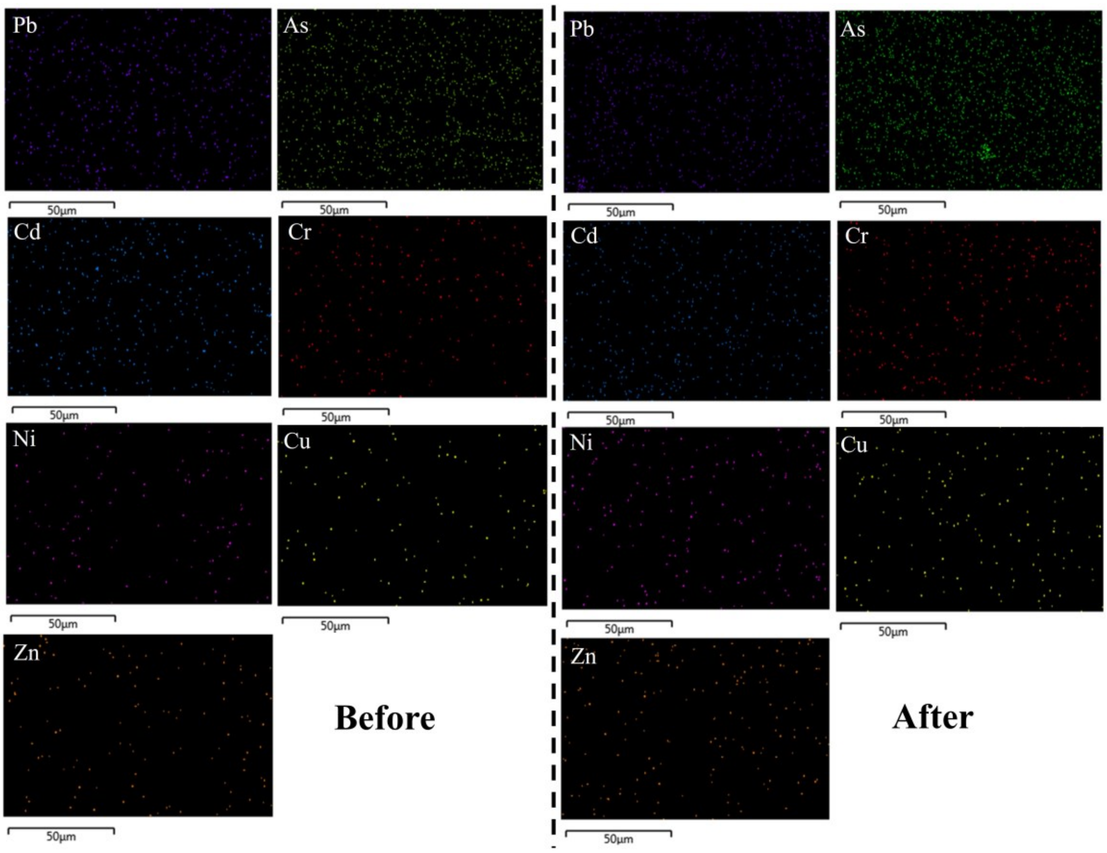

Figure S4. EDS elemental maps of Pb, Zn, Ni, Cr, Cu, As, and Cd on C5S5 before and after adsorption of heavy metals from biogas slurry

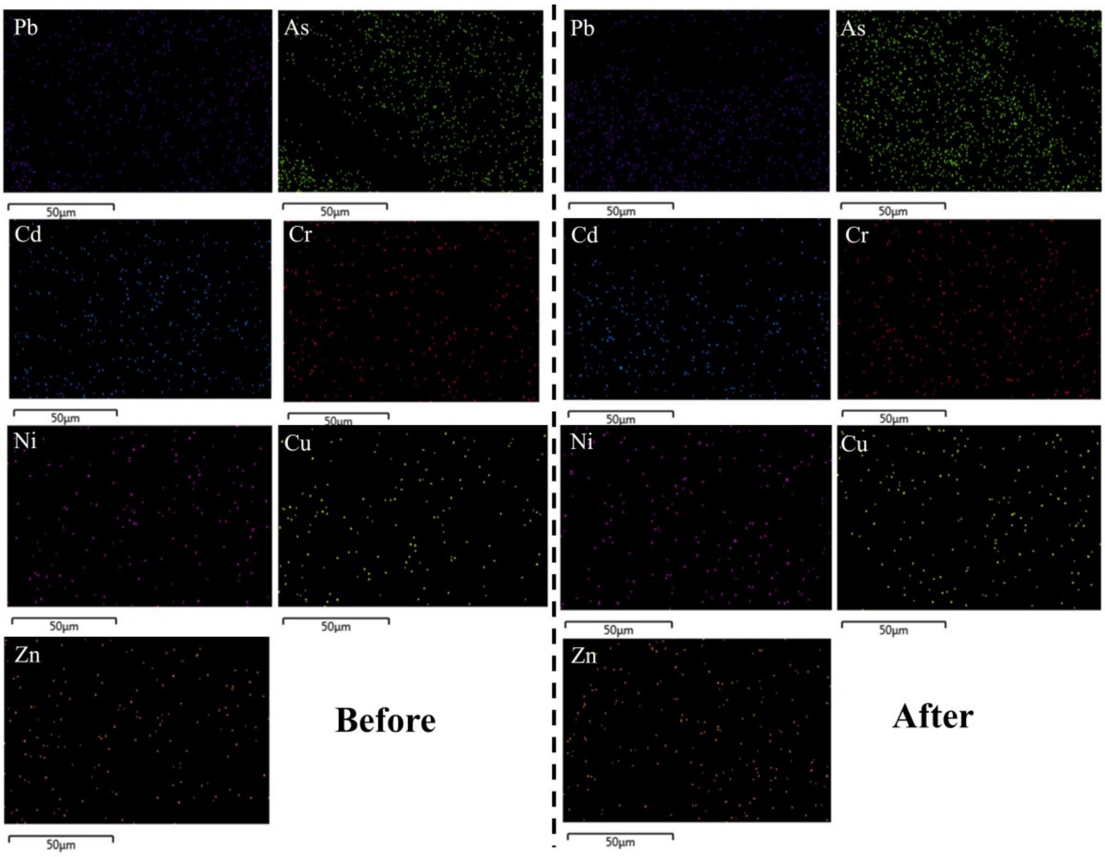

Figure S5. EDS elemental maps of Pb, Zn, Ni, Cr, Cu, As, and Cd on C7S3 before and after adsorption of heavy metals from biogas slurry
